# Retraction: Cecal Lipoma: A Rare Etiology of Acute Appendicitis in Adults

**DOI:** 10.7759/cureus.r95

**Published:** 2024-01-25

**Authors:** Yahya A Alnashri, Aali M Alhuzali, Eyyad A Edrees, Razan A Almuraykhi, Reem A Majrashi, Raghad A Alhomidan, Sarah B Alharbi, Farah A Alassaf, Aishah N Alsuhaibani, Rami S Sufyani, Ahmed S Alkhars, Maisa M Mallawi, Rawan Y Alkhotani, Mustafa M Qattan, Malak Alshammari

**Affiliations:** 1 College of Medicine, Umm Al-Qura University, Mecca, SAU; 2 College of Medicine, Imam Mohammad Ibn Saud Islamic University, Riyadh, SAU; 3 College of Medicine, Najran University, Najran, SAU; 4 College of Medicine, Qassim University, Qassim, SAU; 5 Medicine, King Salman Abdul Aziz Medical City, Madinah, SAU; 6 College of Medicine, Imam Abdulrahman Bin Faisal University, Dammam, SAU; 7 College of Medicine, Ibn Sina National College For Medical Studies, Jeddah, SAU

The Editors-in-Chief have retracted this article. Concerns were raised regarding the identity of the authors on this article. Specifically, Faisal Alhaway and Malak Shammari have stated that they were added to this article without their knowledge or approval. The identity of the other authors could also not be verified. As the appropriate authorship of this work cannot be established, the Editors-in-Chief no longer have confidence in the results and conclusions of this article.

